# Impaired Emotion Recognition after Left Hemispheric Stroke: A Case Report and Brief Review of the Literature

**DOI:** 10.1155/2017/1045039

**Published:** 2017-05-07

**Authors:** Hugo P. Aben, Yael D. Reijmer, Johanna M. A. Visser-Meily, Jacoba M. Spikman, Geert Jan Biessels, Paul L. M. de Kort, PROCRAS Study Group

**Affiliations:** ^1^Department of Neurology, Elisabeth Tweesteden Hospital, Tilburg, Netherlands; ^2^Department of Neurology, Brain Center Rudolf Magnus, University Medical Center Utrecht, Utrecht, Netherlands; ^3^Department of Rehabilitation Medicine, Brain Center Rudolf Magnus, University Medical Center Utrecht, Utrecht, Netherlands; ^4^Department of Clinical and Experimental Neuropsychology, University of Groningen, Groningen, Netherlands

## Abstract

Impaired recognition of emotion after stroke can have important implications for social competency, social participation, and consequently quality of life. We describe a case of left hemispheric ischemic stroke with impaired recognition of specifically faces expressing fear. Three months later, the patient's spouse reports that the patient was irritable and slow in communication, which may be caused by the impaired emotion recognition. The case is discussed in relation to the literature concerning emotion recognition and its neural correlates. Our case supports the notion that emotion recognition, including fear recognition, is regulated by a network of interconnected brain regions located in both hemispheres. We conclude that impaired emotion recognition is not uncommon after stroke and can be caused by dysfunction of this emotion-network.

## 1. Introduction

Social cognition concerns the psychological processes by which we perceive, process, and interpret social information [1]. Nowadays, neurologists are increasingly aware of the importance of screening for deficits in social cognition [2]. For example, screening for impaired social cognition has become more common in patients with traumatic brain injury [2, 3]. After stroke, impaired social cognition is prevalent, with a reported prevalence rate of 49% [4]. Despite the high prevalence, deficits in social cognition after stroke are often overlooked by the neurologist, and it is generally not spontaneously mentioned by the patient or his caregiver.

After stroke, impaired social cognition is partly reflected by worse emotion recognition in studies that compared patients to healthy controls in tasks examining facial, prosodic, and lexical emotional stimuli [5, 6]. These studies show that impaired emotion recognition after stroke is not limited to one modality: a stroke affects general processing of emotion for different modalities.

Impaired emotion recognition after stroke has a negative influence on social participation, and it can have important implications for a patient's quality of life [7]. Moreover, it can affect social competence [8], it predicts social behavior disorders [9], and it is negatively correlated with maintenance of personal and professional relations [10, 11].

We present a case with a profound difficulty in the recognition of faces expressing fear after insular stroke. Furthermore, the case is discussed in relation to the literature concerning emotion recognition and its neural correlates.

## 2. Case Description

**Figure 1:**
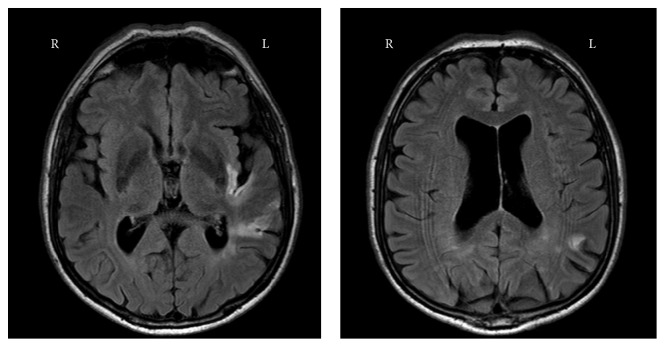
Transversal fluid attenuated inversion recovery (FLAIR) images of the patient, revealing a lesion to the posterior part of the insula and part of the left temporal cortex and left parietal cortex.

**Figure 2:**
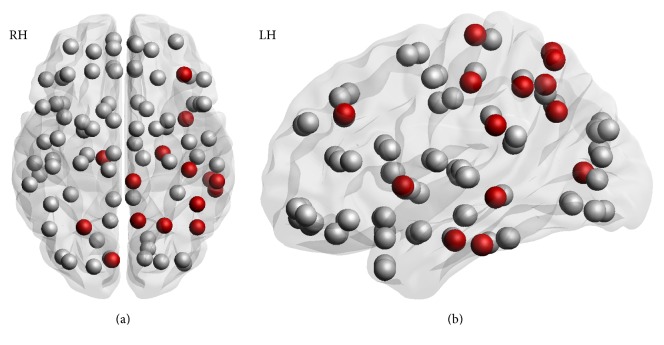
Axial (a) and lateral (b) view of the disconnectivity profile of the patient (obtained from analyzing the DTI-MRI data). Nodes represent 90 cortical and subcortical brain regions [38]. The red nodes indicate regions with more than 1.5 standard deviations of decreased connectivity strength compared to 25 age-matched healthy controls. Note that nodes surrounding the ischemic zone are affected. In addition, however, nodes in the contralesional hemisphere and a frontal node are affected. This suggests that the stroke affected a network of brain regions.

A sixty-five-year-old, right-handed man, who had just retired from his work as a detective, presented with global aphasia, agitation, and a mild paresis of his right arm, which started acutely 5 hours before admission. He had no known vascular risk factors, and he had a degree in higher vocational education. The admission CT scan of his brain revealed no signs of ischemia or hemorrhage. One day after admission, the total score on the Montreal Cognitive Assessment (MoCA) was 20 out of a maximum of 30 points, mainly failing the subdomains language (0/3), delayed recall (2/5), and visuospatial function (2/5). The patient correctly copied the cube and made no mistake in the contour of the clock. However, he did not add numbers to the clock and placed the hands at ten to eleven, instead of ten past eleven. Two days after admission, the patient did not report any complaints. The aphasia, paresis, and agitation had resolved quickly, and the patient was discharged home. As part of an observational study, the PROCRAS study, see Additional Points for more information, the patient underwent a detailed neuropsychological assessment and MRI scan of his brain 4 weeks after the stroke. Apart from complaining of being tired more quickly, he had no other complaints at that point. As part of the neuropsychological assessment, the “Ekman 60 Faces Test,” part of the Facial Expressions of Emotion: Stimuli and Tests (FEEST), was performed [12]. Interestingly, out of 10 presented fearful faces, he recognized only 1 facial expression correctly. For his performance on other emotions, see Table 1. His total score on the FEEST was nearly 3 standard deviations below the norm. There were no symptoms of depression or anxiety on the Hospital Anxiety and Depression Scale (HADS). Moreover, there were no signs of prosopagnosia, and the Bells Test revealed no visuospatial dysfunction. Further extensive neuropsychological testing revealed problems with naming objects on the Boston Naming Test but no impaired performance on the verbal fluency test. In Table 2, the *T*-scores for each domain and the subtests that constitute these domains are presented. The MRI showed a lesion of the posterior part of the left insula but also of a part of the left temporal cortex and the left parietal cortex as can be seen in Figure 1. In order to investigate potential impairments in brain connectivity, the brain network of the patient was reconstructed from the diffusion weighted MR images as described in Reijmer et al. [13]. The mean fractional anisotropy of the white matter tracts connected to each of the 90 cortical and subcortical brain regions was calculated. The brain networks of 25 age-matched healthy control participants served as a reference group. Results showed that the connectivity strength of the patient was markedly reduced (>1.5 SD lower compared to the reference sample) in several parietal, temporal, and subcortical regions, primarily in the left hemisphere (Figure 2), indicating disconnection of a network of brain regions.

Three months after the stroke, the patient felt no restrictions in his ability to perform hobbies such as gardening and reading, and he experienced no difficulties in social contact. However, his wife reported that he has been slower in communication and manifests slight irritability ever since the stroke.

## 3. Discussion

In this article, we present a case with a profound deficit in the recognition of facial emotions expressing fear after enduring a stroke of the posterior part of the left insula and parts of the left temporal and parietal cortex. There was no evidence of depressive or anxious symptoms on follow-up, suggesting that the difficulty in recognition of fearful faces was not caused by a depressive or anxiety disorder. It has not been ruled out that visuospatial dysfunction caused the impaired recognition of facial emotions. However, the score of 2/5 for the MoCA's visuospatial subdomain might have been caused by a language disorder rather than a visuospatial disorder. Moreover, the Bells Test revealed no abnormalities at the neuropsychological examination 4 weeks after stroke, making a neglect very unlikely. It was not clear whether our patient had difficulties in emotion recognition before the stroke, for example, due to an autism spectrum disorder or traumatic brain injury in the past. However, we think that his higher education level and his work as a detective require at least adequate social cognitive skills. Therefore, it is plausible to attribute the profound deficit in fear recognition to the stroke. This case stood out because of two reasons. First, the stroke involved the left hemisphere, while emotion recognition is generally attributed to the right hemisphere [5]. Secondly, the deficit in recognition of fearful faces after ischemia of the posterior part of the insula seems to contradict earlier findings in the literature [14–16]. Recognition of fear has been strongly linked to activity in the amygdala [16], whereas the insula is thought to be specifically engaged in the recognition of disgust [14, 15]. We will discuss the literature in relation to the case and these two findings.

### 3.1. Laterality in Emotion Recognition

Researchers have still not reached consensus on the specific contribution of the right and left hemisphere to emotion recognition. A recent review of many studies examining emotion recognition after stroke found more support for the hypothesis that emotion recognition seems to be largely lateralized to the right hemisphere, independent of valence [5, 17]. Several studies report that left hemispheric stroke patients perform as good as healthy controls in the recognition of emotions [18–21]. However, other studies do reveal differences in emotion recognition between patients with a left hemispheric stroke and healthy controls [22–24]. The left hemispheric stroke of our patient is in line with these studies and adds to the theory that the left hemisphere also contributes to the process of emotion recognition. According to the motoric direction theory, the left hemisphere is specialized in “approach” emotions (i.e., happiness, anger, and surprise), whereas the right hemisphere is responsible for recognizing “withdrawal” emotions (i.e., sadness, fear, and disgust) [25]. The patient had relative ease in recognizing anger and happiness, although he scored below the cutoff score for surprise. He scored relatively low for the withdrawal emotions: fear, sadness, and disgust. Although the patient does partly fit the pattern of this hypothesis, the location of the stroke would suggest that the specialization of both hemispheres is just the opposite of what the motoric direction theory suggests. Possibly, disconnectivity between both hemispheres (Figure 2) could explain this mismatch.

### 3.2. Neural Correlates of Emotion Recognition

Several brain structures have been suggested to be involved in emotion recognition. For example, lesions to left or right temporal cortex lead to impaired emotion recognition [26]. However, lesions to the basal ganglia [27, 28], the cerebellum [29], the thalamus [30], the right inferior parietal cortex, and the anterior infracalcarine cortex [18] can have the same consequence. It is well known that activity in the amygdala has been strongly linked to recognition of fear [16], and a lesion of the amygdala causes an impairment in fear recognition [31–33]. However, this does not imply that lesions of the amygdala are the unique cause of impaired fear recognition. For example, a study reported that a lesion to the right infracalcarine cortex also seems to lead to a specific impairment in the recognition of fear [18]. This suggests that even the recognition of one single emotion, in this case the emotion fear, is a process that involves, like all cognitive functions [34], a network of brain structures.

A meta-analysis of fMRI and PET studies [16] in healthy controls concluded that the neural network involved in recognition of emotion consists of three important structures. The first is the medial prefrontal cortex (MPFC), which has a general role in emotional processing. The other two are the anterior cingulate cortex (ACC) and the insula, which are more specifically involved in emotion recognition, especially in more cognitive demanding tasks [16].

### 3.3. The Insula and Emotion Recognition

Because our patient had a stroke involving, among others, the posterior part of the left insula, this specific lesion might have caused the difficulty in recognition of emotion. In behavioral and imaging studies, the insula is found to be engaged in the recognition of disgust [14, 15]. However, less is known about the consequences of a lesion to the insula. Several case studies describe different responses to an acute lesion of the left insula. In one study, a patient had impairments in global cognitive functioning as reflected by cognitive screening with the MoCA [35], comparable with the case we described. In a double case report, one patient with insular stroke had only slightly slower response times but no impaired emotion recognition [36]. The second case that is described had a subcortical stroke, disconnecting the connections from the insula to the frontal cortex. In this case, an impaired recognition of all negative emotions was found. Another study reported on a patient with behavioral changes after an isolated stroke of the anterior part of the right insula [37]. Although emotion perception was not assessed, an additional SPECT scan showed hypoperfusion in several right anterior brain structures, most of which are thought to be involved in an emotion-network.

### 3.4. A Matter of (Dis)connection?

As stated earlier, the left insula plays a role in a network of structures involved in emotion recognition. This can be supported by a recent study in which direct electrical stimulation was applied to the left insula during awake surgery in thirteen patients causing a decreased recognition of all negative emotions [39]. Another study found that impaired emotion recognition was associated with dysfunction of a bilateral fronto-temporal-limbic network in 180 patients with traumatic brain injury (TBI) [40]. A different study found that damage anywhere to the inferior frontooccipital fasciculus or the inferior longitudinal fasciculus is associated with impaired emotion recognition [41]. The seemingly contradicting evidence of different presentations after insular stroke can be better interpreted as the consequence of disconnection to frontal or a variety of contralateral regions [42]. This implies that the process of emotion recognition is not confined to a specific location, a lesion anywhere in this “emotion-network” may lead to a deficit in emotion recognition [43].

We hypothesize that in our patient the stroke caused damage to the network involved in emotion recognition, which is supported by the analysis of the DTI-MRI data (Figure 2). Although this analysis does not provide direct evidence for emotion recognition being a network-function, it does support this theory by showing disconnection in more brain structures than those directly affected by the stroke. We propose that the profound impairment of recognition of fear can be explained by a disconnection of fibers to this emotion-network.

### 3.5. Impact of Impaired Emotion Recognition after Stroke

Impaired emotion recognition has been found to negatively impact social participation and quality of life after stroke [44]. For deficits in emotion regulation, the same consequences have been established [45]. The patient's spouse reported that the patient showed slight irritability and slowness in communication after the stroke, although the patient himself stated having no difficulties in social interaction. We hypothesize that the deficit in emotion recognition could partly contribute to these symptoms, and this deficit could consequently lead to restrictions in social participation.

## 4. Conclusions

Impaired emotion recognition is prevalent after stroke. However, it is not often reported by the patient or the caregiver, it is not easily recognized, and it is not routinely investigated in clinical practice. This is unfortunate, since impaired emotion recognition negatively impacts social participation. Detection of impaired emotion recognition may help in guidance of the patient and his caregiver. In turn, this should help people better reintegrate into daily life and increase quality of life.

Emotion recognition is a complex process involving many neural structures. A lesion anywhere in this emotion-network can lead to dysfunction. Further research on emotion recognition should shift its focus from the specific localization of emotion recognition to identifying the brain regions and the functional and structural connections that form part of this emotion-network.

## Figures and Tables

**Table 1:** The patient's results for the “Ekman 60 Faces Test.”

Emotion	Nr correct answers	Mean scores reference sample (SD)	Cutoff values
Anger	8/10	7.33 (1.90)	4
Disgust	6/10	9.00 (1.62)	6
Fear	1/10^*∗*^	6.47 (2.03)	3
Happiness	10/10	9.93 (0.42)	9
Sadness	5/10	8.03 (1.66)	5
Surprise	5/10^*∗*^	8.66 (1.44)	6

Total	35/60^*∗*^	49.41 (4.88)	41

The mean scores, standard deviations. and cutoff values for the reference sample (aged 61–70) are derived from the FEEST manual. This sample consisted of 58 healthy participants with an IQ > 90. Cutoff values have been calculated by using the nearest integer value to a *z*-value of 1.65 below average [12]. ^*∗*^Scores below the cutoff value.

**Table 2:** Neuropsychological assessment of the patient.

Domain	*T*-score	Tests	*T*-scores
Attention and processing speed	46.6	Reaction Time Test, Vienna Test System, S1, S2	50.5
		Symbol Digit Modalities Test	44.2
		Trail Making Test A	41.0

(Working) memory and learning	39.2	WAIS Digit Span Forward and Backward	41.7
		The Rey Auditory Verbal Learning Test	38.0

Frontal-Executive functions	39.8	Controlled Oral Word Association Test	30.0^*∗*^
		Hayling Test	40.0
		Reaction time test, Vienna Test System, S3	47.0
		Trail Making Test B	44.0

Language	31.6^*∗*^	Boston Naming Test	23.1^*∗*^
		Semantic fluency	40.0

Visuospatial function	NA	Bells Test, no *T*-score available, 1 omission	NA

This table shows the *T*-scores of the patient for each of the tested domains in the neuropsychological assessment. ^*∗*^Scores below 1.5 standard deviations.

## Additional Points

In the Prediction of Cognitive Recovery After Stroke (PROCRAS) study, we aim to investigate the additional value of measures of structural connectivity derived from diffusion tensor imaging- (DTI-) MRI in the prediction of cognitive recovery after stroke. Patients with the clinical diagnosis of a stroke with a MoCA total score below 26 undergo a neuropsychological assessment and DTI-MRI 3–6 weeks after stroke. One year after stroke the neuropsychological assessment is repeated to assess the primary endpoint.

## References

[1] Henry J. D., von Hippel W., Molenberghs P., Lee T., Sachdev P. S. (2016). Clinical assessment of social cognitive function in neurological disorders. *Nature Reviews Neurology*.

[2] Benedictus M. R., Spikman J. M., Van Der Naalt J. (2010). Cognitive and behavioral impairment in traumatic brain injury related to outcome and return to work. *Archives of Physical Medicine and Rehabilitation*.

[3] Spikman J. M., Timmerman M. E., Milders M. V., Veenstra W. S., van der Naalt J. (2012). Social cognition impairments in relation to general cognitive deficits, injury severity, and prefrontal lesions in traumatic brain injury patients. *Journal of Neurotrauma*.

[4] Starkstein S. E., Leiguarda R. C., Federoff J. P., Price T. R., Robinson R. G. (1994). Neuropsychological and neuroradiologic correlates of emotional prosody comprehension. *Neurology*.

[5] Yuvaraj R., Murugappan M., Norlinah M. I., Sundaraj K., Khairiyah M. (2013). Review of emotion recognition in stroke patients. *Dementia and Geriatric Cognitive Disorders*.

[6] Borod J. C., Obler L. K., Erhan H. M. (1998). Right hemisphere emotional perception: evidence across multiple channels. *Neuropsychology*.

[7] Kim K., Kim Y. M., Kim E. K. (2014). Correlation between the activities of daily living of stroke patients in a community setting and their quality of life. *Journal of Physical Therapy Science*.

[8] Langer S. L., Pettigrew L. C., Wilson J. F., Blonder L. X. (1998). Personality and social competency following unilateral stroke. *Journal of the International Neuropsychological Society*.

[9] Narme P., Roussel M., Mouras H., Krystkowiak P., Godefroy O. (2016). Does impaired socioemotional functioning account for behavioral dysexecutive disorders? Evidence from a transnosological study. *Aging, Neuropsychology, and Cognition*.

[10] Yeates G., Rowberry M., Dunne S. (2016). Social cognition and executive functioning predictors of supervisors' appraisal of interpersonal behaviour in the workplace following acquired brain injury. *NeuroRehabilitation*.

[11] Blonder L. X., Pettigrew L. C., Kryscio R. J. (2012). Emotion recognition and marital satisfaction in stroke. *Journal of Clinical and Experimental Neuropsychology*.

[12] Young A., Perrett D., Calder A., Sprengelmeyer R., Ekman P. (2002). *Facial Expressions of Emotion—Stimuli and Tests (FEEST)*.

[13] Reijmer Y. D., Leemans A., Caeyenberghs K., Heringa S. M., Koek H. L., Biessels G. J. (2013). Disruption of cerebral networks and cognitive impairment in Alzheimer disease. *Neurology*.

[14] Krolak-Salmon P., Hénaff M.-A., Isnard J. (2003). An attention modulated response to disgust in human ventral anterior insula. *Annals of Neurology*.

[15] Adolphs R., Tranel D., Damasio A. R. (2003). Dissociable neural systems for recognizing emotions. *Brain and Cognition*.

[16] Phan K. L., Wager T., Taylor S. F., Liberzon I. (2002). Functional neuroanatomy of emotion: a meta-analysis of emotion activation studies in PET and fMRI. *NeuroImage*.

[17] Borod J. C. (1992). Interhemispheric and intrahemispheric control of emotion: a focus on unilateral brain damage. *Journal of Consulting and Clinical Psychology*.

[18] Adolphs R., Damasio H., Tranel D., Damasio A. R. (1996). Cortical systems for the recognition of emotion in facial expressions. *Journal of Neuroscience*.

[19] Bowers D., Bauer R. M., Coslett H. B., Heilman K. M. (1985). Processing of faces by patients with unilateral hemisphere lesions. I. Dissociation between judgments of facial affect and facial identity. *Brain and Cognition*.

[20] Charbonneau S., Scherzer B. P., Aspirot D., Cohen H. (2003). Perception and production of facial and prosodic emotions by chronic CVA patients. *Neuropsychologia*.

[21] Schmitt J. J., Hartje W., Willmes K. (1997). Hemispheric asymmetry in the recognition of emotional attitude conveyed by facial expression, prosody and propositional speech. *Cortex*.

[22] Kucharska-Pietura K., Phillips M. L., Gernand W., David A. S. (2003). Perception of emotions from faces and voices following unilateral brain damage. *Neuropsychologia*.

[23] Mandal M. K., Borod J. C., Asthana H. S., Mohanty A., Mohanty S., Koff E. (1999). Effects of lesion variables and emotion type on the perception of facial emotion. *Journal of Nervous and Mental Disease*.

[24] Peper M., Irle E. (1997). The decoding of emotional concepts in patients with focal cerebral lesions. *Brain and Cognition*.

[25] Davidson R. J., Ekman P., Saron C. D., Senulis J. A., Friesen W. V. (1990). Approach-withdrawal and cerebral asymmetry: emotional expression and brain physiology I. *Journal of Personality and Social Psychology*.

[26] Xi C., Zhu Y., Zhu C., Song D., Wang Y., Wang K. (2013). Deficit of theory of mind after temporal lobe cerebral infarction. *Behavioral and Brain Functions*.

[27] Kemp J., Berthel M.-C., Dufour A. (2013). Caudate nucleus and social cognition: neuropsychological and SPECT evidence from a patient with focal caudate lesion. *Cortex*.

[28] Cheung C. C. Y., Lee T. M. C., Yip J. T. H., King K. E., Li L. S. W. (2006). The differential effects of thalamus and basal ganglia on facial emotion recognition. *Brain and Cognition*.

[29] Adamaszek M., Kirkby K. C., Dagata F. (2015). Neural correlates of impaired emotional face recognition in cerebellar lesions. *Brain Research*.

[30] Wilkos E., Brown T. J. B., Slawinska K., Akucharska K. (2015). Social cognitive and neurocognitive deficits in inpatients with unilateral thalamic lesions—pilot study. *Neuropsychiatric Disease and Treatment*.

[31] Adolphs R., Gosselin F., Buchanan T. W., Tranel D., Schyns P., Damasio A. R. (2005). A mechanism for impaired fear recognition after amygdala damage. *Nature*.

[32] Bach D. R., Hurlemann R., Dolan R. J. (2015). Impaired threat prioritisation after selective bilateral amygdala lesions. *Cortex*.

[33] Dellacherie D., Hasboun D., Baulac M., Belin P., Samson S. (2011). Impaired recognition of fear in voices and reduced anxiety after unilateral temporal lobe resection. *Neuropsychologia*.

[34] Park H.-J., Friston K. (2013). Structural and functional brain networks: from connections to cognition. *Science*.

[35] Julayanont P., Ruthirago D., DeToledo J. C. (2016). Isolated left posterior insular infarction and convergent roles in verbal fluency, language, memory, and executive function. *Proceedings (Baylor University Medical Center)*.

[36] Couto B., Sedeño L., Sposato L. A. (2013). Insular networks for emotional processing and social cognition: comparison of two case reports with either cortical or subcortical involvement. *Cortex*.

[37] Cho H.-J., Kim S.-J., Hwang S. J. (2012). Social-emotional dysfunction after isolated right anterior insular infarction. *Journal of Neurology*.

[38] Tzourio-Mazoyer N., Landeau B., Papathanassiou D. (2002). Automated anatomical labeling of activations in SPM using a macroscopic anatomical parcellation of the MNI MRI single-subject brain. *NeuroImage*.

[39] Papagno C., Pisoni A., Mattavelli G. (2016). Specific disgust processing in the left insula: new evidence from direct electrical stimulation. *Neuropsychologia*.

[40] Dal Monte O., Krueger F., Solomon J. M. (2013). A voxel-based lesion study on facial emotion recognition after penetrating brain injury. *Social Cognitive and Affective Neuroscience*.

[41] Philippi C. L., Mehta S., Grabowski T., Adolphs R., Rudrauf D. (2009). Damage to association fiber tracts impairs recognition of the facial expression of emotion. *Journal of Neuroscience*.

[42] Limongi R., Tomio A., Ibanez A. (2014). Dynamical predictions of insular hubs for social cognition and their application to stroke. *Frontiers in Behavioral Neuroscience*.

[43] Kennedy D. P., Adolphs R. (2012). The social brain in psychiatric and neurological disorders. *Trends in Cognitive Sciences*.

[44] Cooper C. L., Phillips L. H., Johnston M., Radlak B., Hamilton S., McLeod M. J. (2014). Links between emotion perception and social participation restriction following stroke. *Brain Injury*.

[45] Cooper C. L., Phillips L. H., Johnston M., Whyte M., Macleod M. J. (2015). The role of emotion regulation on social participation following stroke. *British Journal of Clinical Psychology*.

